# Supplementary figures and images for: Interfering with the ERC1–LL5β interaction disrupts plasma membrane–Associated platforms and affects tumor cell motility
Source: PLoS One. 2023 Jul 12;18(7):e0287670. doi: 10.1371/journal.pone.0287670 (PMC10337942; doi:10.1371/journal.pone.0287670)

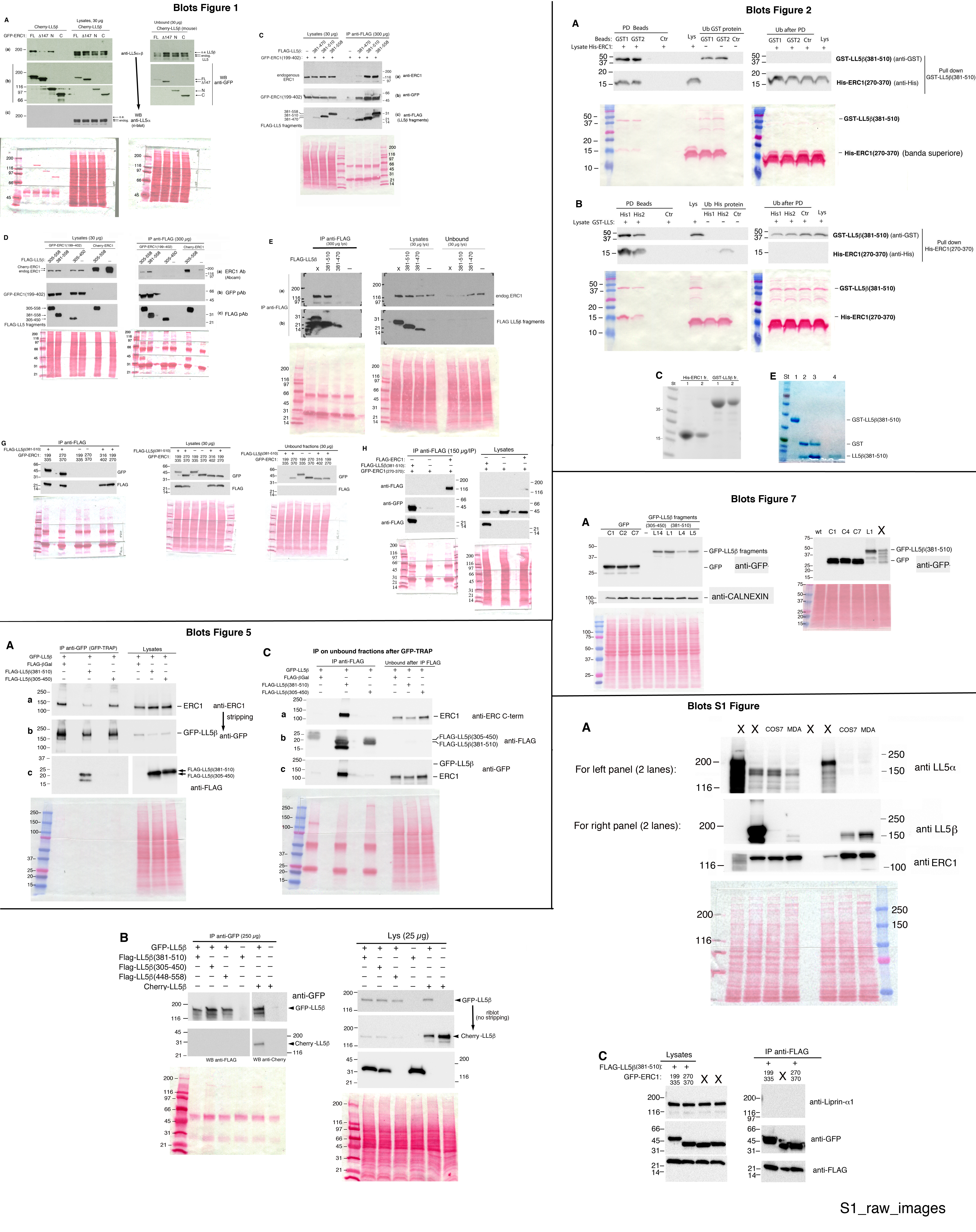

Supplement: S1 Raw images — Unedited images of the blots and gels shown in the indicated panels of Figs 1, 2, 5, 7, and S1 Fig. The description of the experimental conditions are described in the legends of the respective Figures. (TIF) [file pone.0287670.s001.tif]

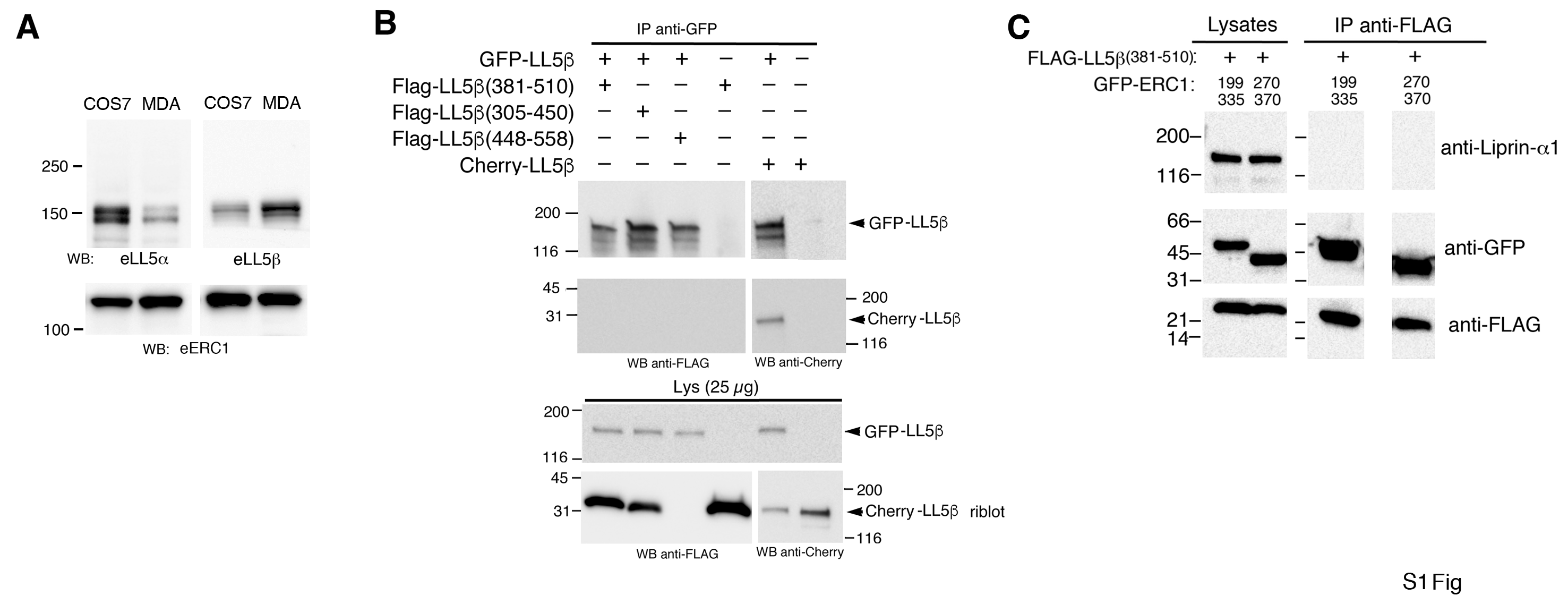

Supplement: S1 Fig — (A) Aliquots of lysates (30 μg protein) from COS7 and MDA-MB-231 cells before (left filters) or after (right filters) immunoprecipitation of endogenous LL5α were blotted with the indicated specific antibodies to reveal endogenous LL5α, LL5β or ERC1. (B) Aliquots (250 μg protein) of lysates from COS7 cells co-transfected with the indicated constructs were used for immunoprecipitation with anti-GFP Abs. Membranes were cut and immunostained with the indicated Abs. (C) Aliquots (150 μg protein) of lysates from COS7 cells transfected with GFP–tagged ERC1 and FLAG-LL5β(381–510) constructs were immunoprecipitated with anti-FLAG Abs. Immunoprecipitates (IP) and lysates (30 μg) were immunoblotted to detect the tagged constructs and endogenous Liprin-α1. (TIF) [file pone.0287670.s002.tif]

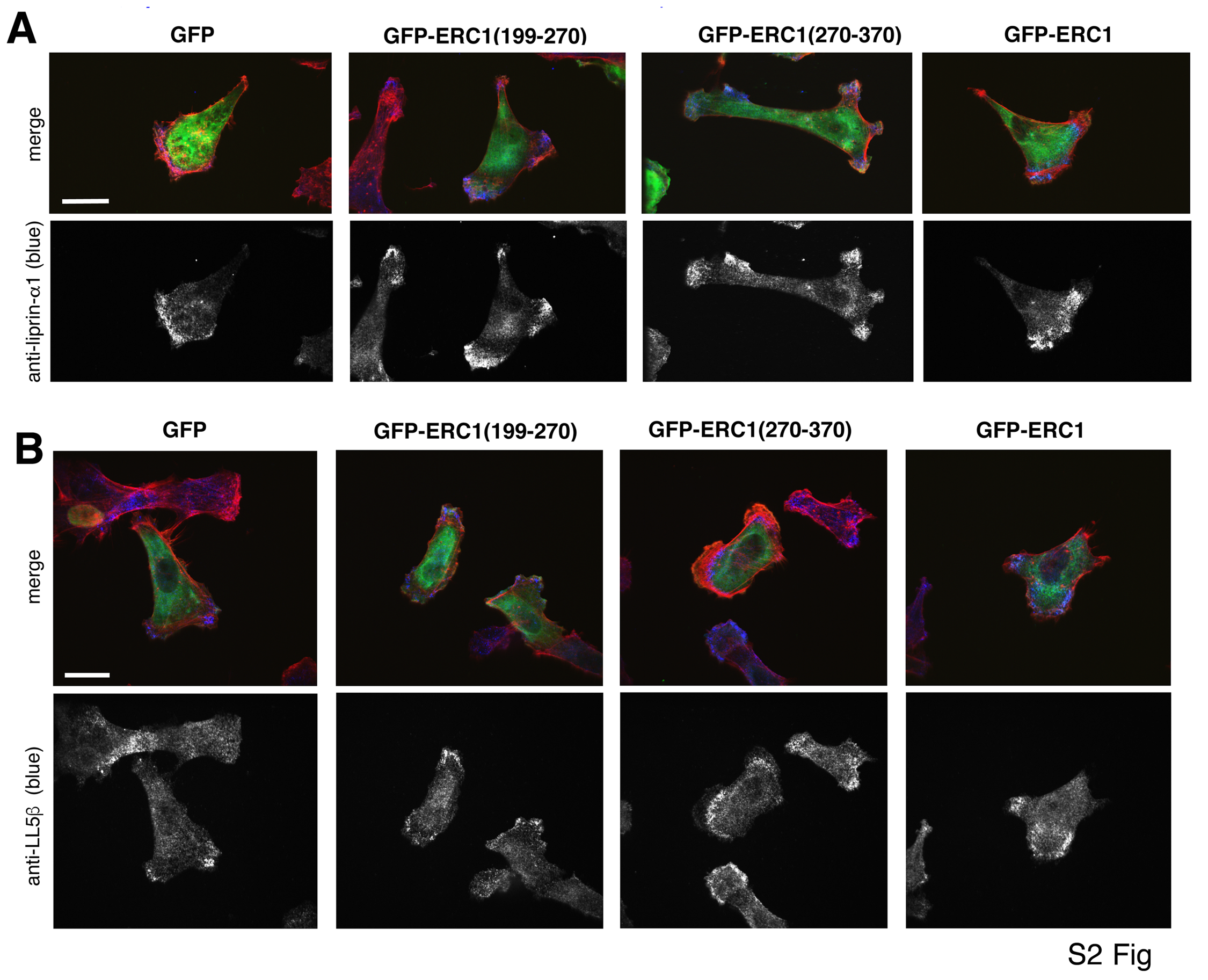

Supplement: S2 Fig — Localization of endogenous Liprin-α1 (A) and LL5 proteins (B) in migrating MDA-MB-231 cells transfected with the indicated constructs. Bars, 20 μm. (TIF) [file pone.0287670.s003.tif]

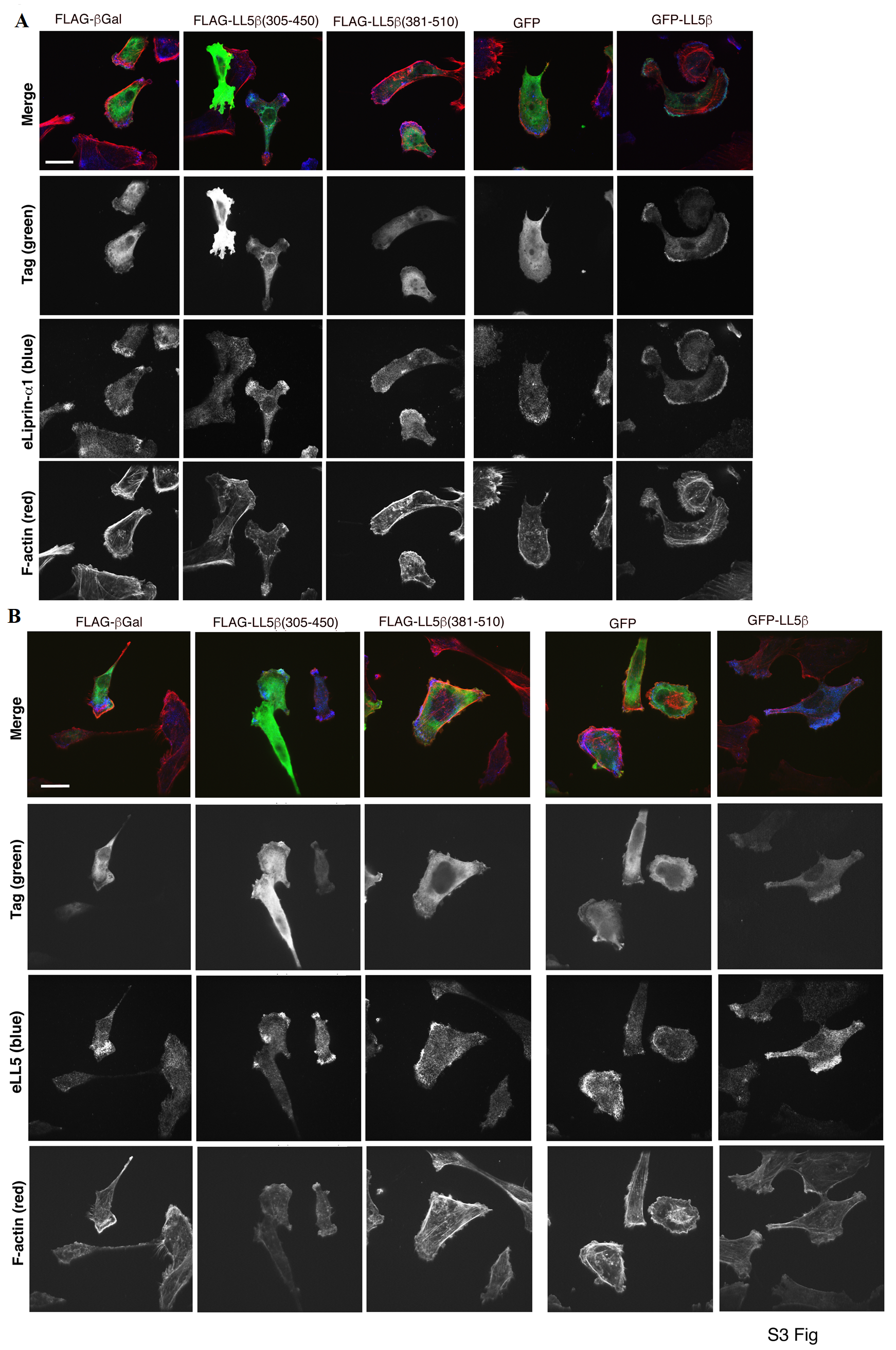

Supplement: S3 Fig — Localization of endogenous Liprin-α1 (A) and LL5 proteins (B) in migrating MDA-MB-231 cells transfected with the indicated constructs. Bars, 20 μm. (TIF) [file pone.0287670.s004.tif]

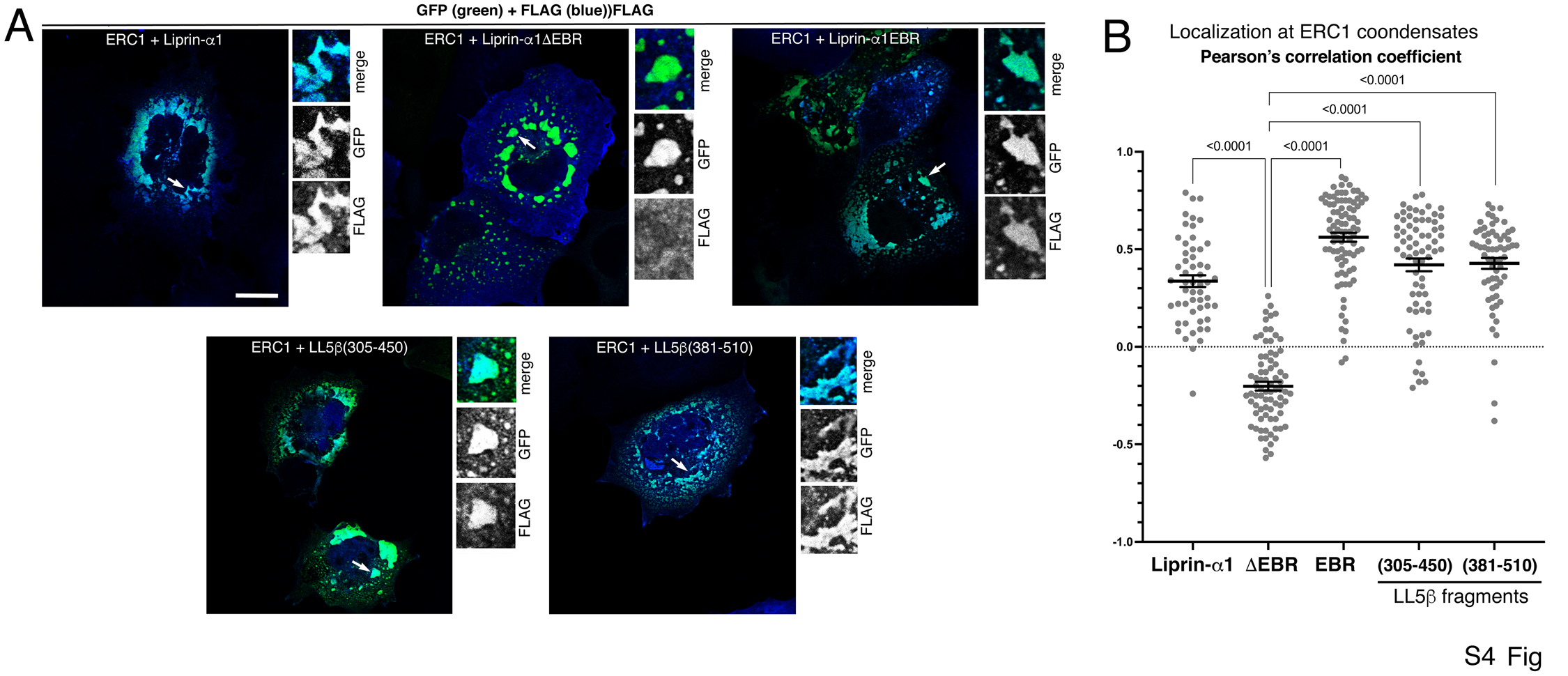

Supplement: S4 Fig — (A) Specific recruitment of FLAG-tagged (blue) LL5β fragments at GFP-ERC1 (green) condensates in co-transfected COS7 cells. Bar = 20 μm. Three-fold enlargements of areas indicated by arrows. (B) Pearson’s correlation coefficient for the colocalization of fragments at ERC1 condensates. Mean ±SEM; n = 54–89 condensates analyzed for each condition from 3 experiments. ANOVA, Tukey post hoc; FLAG-Liprin-α1-ΔEBR as negative control. (TIF) [file pone.0287670.s005.tif]

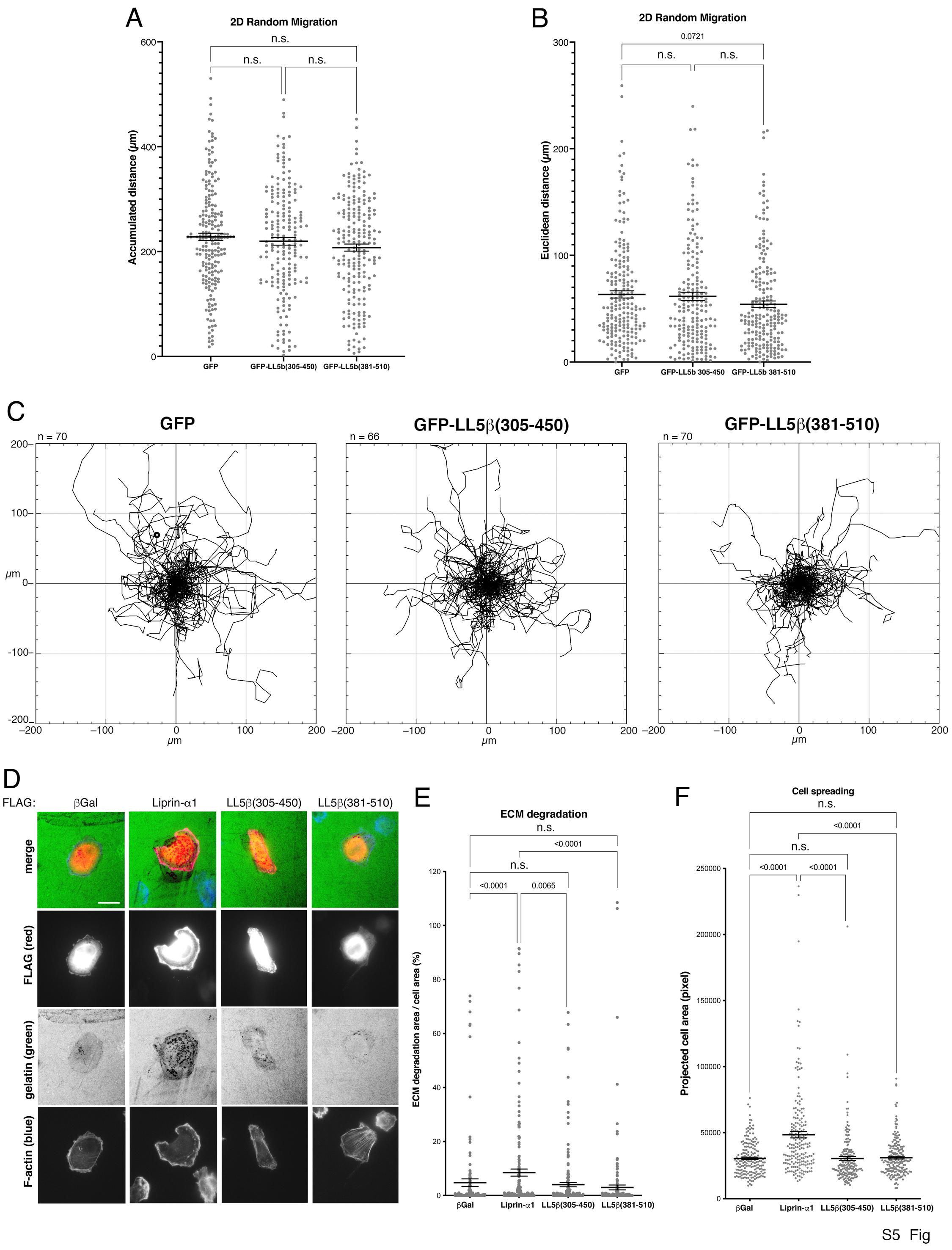

Supplement: S5 Fig — (A-B) 2D random cell migration: accumulated distance (A) and Euclidean distance (B) of transfected MDA-MB-231 cells plated on 2.5 μg/ml FN and tracked for 5 h. Mean ±SEM; n = 194–208 cells from 3 experiments. One way ANOVA, Kruskal-Wallis test, Dunn’s multiple comparisons. (C) Trajectories of MDA-MB-231 cells transfected with the indicated constructs, plated in NIH-3T3-derived 3D extracellular matrix, and tracked for 8 h (n = 81–84 cells per experimental condition). (D-F) LL5β(381–510) does not affect extracellular matrix degradation. Transfected MDA-MB-231 cells were plated on fluorescently labelled gelatin. After 5 hours the dark areas of gelatin degradation (D; bar, 20 μm) were quantified (E). For each cell analyzed, the gelatin degradation area was normalized to the corresponding projected cell area (F). Mean ±SEM; n = 186–194 cells from 4 experiments. One way ANOVA, Kruskal-Wallis test, Dunn’s multiple comparisons. (TIF) [file pone.0287670.s006.tif]

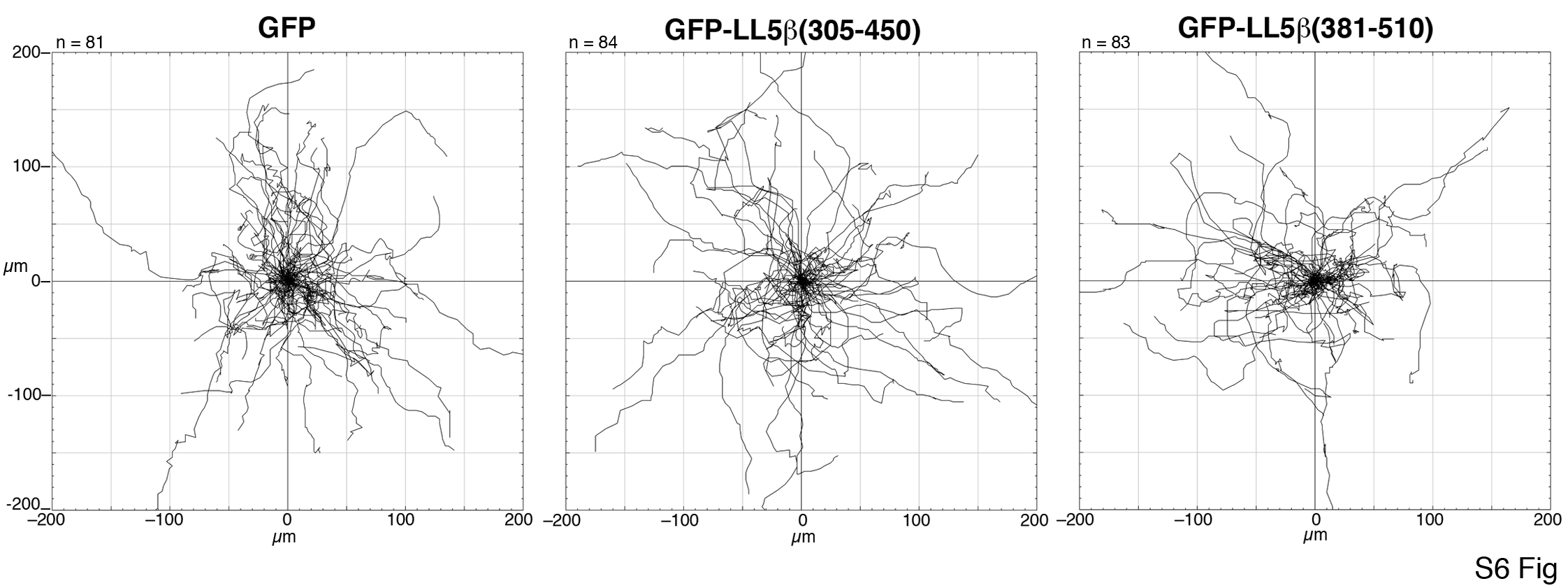

Supplement: S6 Fig — Trajectories of MDA-MB-231 cells transfected with the indicated constructs, plated in NIH-3T3-derived 3D extracellular matrix, and tracked for 8 h (n = 81–84 cells per experimental condition). (TIF) [file pone.0287670.s007.tif]
